# Control of Food and Other Prices

**Published:** 1918-01

**Authors:** 


**A Monthly Review of Medicine and Surgery**

EDITOR AND PUBLISHER, DR. A. L. BENEDICT, 228 Summer St., corner of Elmwood Ave., Buffalo, (Address for all communications. Please make personal and telephone calls before 1 P. M.)

Third, in age, on the western continent. Independent, but supports professional organizations. News limited mainly to Western N. Y. and Penn. Subscription $2.00 a year; $3.00 for two years in advance. Changes and errors in address or failure or duplication of delivery, should be reported immediately.

Yearly Volume 73 JANUARY, 1918 Number 6

### Control of Food and Other Prices.

The control of food prices is the greatest necessity along the lines of paternal government, especially in war. But, in a complicated civilization, the ultimate and obvious reason is not the correct one for practical logic. In such a civilization, the individual or family, has an income which must be distributed, in dollars and cents, among various necessities and, down to the actual requirements of nutrition, food is, from many aspects, the least necessary. It is easy enough to say that this statement is absurd and to support the objection on physiologic and anthropologic grounds. But, from the standpoint of the typic civilized individual or family, far removed from the direct problem as to whether he shall use his time and strength in catching a rabbit or gathering seeds and berries, or in building a hut or weaving a blanket, or making a fire place, or making beads to decorate himself, with all sorts of necessities, comforts and luxuries equally available providing he has the money to buy them, the hard fact is that he gets less psychic gratification from food than from almost anything else and that, therefore, he is more disposed to scrimp on food than anything else. There are numerous exceptions to support the idea of the prime necessity of food but they are not so much types of modern civilization as survivals of the savage in a civilized population. It must also be remembered that a good deal of extravagance in the way of food is really not so much in the purchase of nutriment as of music, cabaret shows and dancing. Also, it must be remembered that we charge to food a good deal that is precisely analogous to the time and labor that the savage spent in making beads and that even the savage spent a good deal of his energy on this last and similar desiderata.

The real, practical, present reason for emphasizing control of food prices is that at the lowest wage limit, the individual must spend about half his earnings on table board (which is not so much a tautology as it appears) and the family on food stuffs. Double this actual expense is enough for anyone to spend on food and, however much the actual food expense increases, it very rapidly declines to 10% of the total income for the fairly well-to-do family and to a percentage so small for the rich that no conceivable increase in food prices can affect them appreciably.

Now, while the indirect tax of war will, if not opposed by government paternalism, fall heaviest on the very poor, the direct tax which amounts to about $110. per capita per annum, according to present estimates, can not be limited to the rich but will apply to a very considerable degree to the fairly well-to-do. Moreover the indirect, economic tax will, relatively, affect this class almost as much so far as mental worry and deprivation of the comforts and luxuries, including spiritual and intellectual as well as physical luxuries, as it will affect the very poor. Even if we say they should worry, we must not overlook the fact that, by direct employment, indirect support through wages and various philanthropies, the well-to-do class, probably does more to support the very poor than the rich, considering the enormous numeric difference.

Just how much importance have food prices for the well-to-do, meaning families with incomes of $2000-$5000? Actual and proportionate distributions of family budgets vary widely, largely according to size of family but almost equally according to preferences for one or another means of comfort of ostentation. Approximately, it may be said that for every dollar spent for food, such families spend 25 to 40 cents for fuel, 25 to 75 cents for domestic wages, 50 cents for clothing for the man, 75 for the woman and 50 for the children; one to two dollars for rent, or its equivalent; 10 to 25 cents for public commodities, light, water, telephone, etc.; 50 cents to two dollars for recreation and amusement including travel and, therefore, railroad fares; and a dollar and a half for automobile, which has, for many, reduced the former item from what would have been considered normal a decade ago. These total somewhere from seven to ten dollars and may, on a very approximate basis, be considered daily expenses.

However these estimates may differ from the actual for a particular family, the fact remains that food is a comparatively small item of the total and one that, beyond actual physiologic needs and reasonable enjoyment, is not so strongly demanded as any one of the others. Even with regard to food itself, it makes no difference where a saving is affected. Most of these general subdivisions of the budget and most of the items in each, have increased 10-50% as compared with a generation ago and some have increased as much as the direct results of war. However, if we allow for direct and explainable increases due to war, an actual study of prices, shows that the pessimistic view of the increase of cost of living is not justified for a great many articles and we must admit, in general, that, barring immediate disturbances due to war, there has never been a time or place in the world's history where the average individual's labor produced so much in comfort as now and here.

When we think of control of food prices, the first notion, is that we are dealing with a unit. As a matter of fact, this notion is absolutely incorrect. We are dealing with laborers whose wages we certainly do not want to diminish, with small capitalists and producers (farmers, etc.) who are not earning unreasonable returns for their investment and personal labor, with speculators and trusts. On the whole, none of these classes engaged in food production and distribution can have their earnings and profits attacked in the interests of the public, with so much justice and freedom from hardship as those engaged in providing other commodities and services. For many kinds of foods, marine or land transportation is the main point at which to direct efforts toward lower prices. In other cases, customs duties must be changed and the tax directed in other ways. For milk, we must consider glass and fibre containers; for preserved vegetables and fruits, tinware, rather than the actual cost of production of the food itself. Any one can follow the line of argument for any particular food stuff and he will be surprised at the complexity of the general problem and at its tendency to go off on a tangent along a line that has nothing to do with food directly and that leads into all sorts of other considerations. For example, many kinds of foods can be materially reduced in price only by cheapening transportation. If we reduce to the standard of Europe or of certain parts of this country or hold against the advance now demanded, the rates of transportation for human beings, we can save for the average well-to-do individual (man, woman or child) as much or more than his yearly ration of a barrel of flour. If we limit the discussion to transportation after discharge from the railroad terminal or before it is reached, we find that, on any fairly large scale, carriage by gasoline motor is conceded to be the most economic and that the principal items on which a saving can be effected are gasoline and tires. The former has advanced about 150%, the latter about 50%, the former without any real justification. But, if we compel the reduction of price of gasoline to the normal for the sake of hauling crackers and flour and vegetables, we will save the ultimate consumer of the foods approximately 15 cents a gallon on an average of 500 gallons a year—$75. Right here, we have saved him about 20% of his entire food expense, not to mention the enormous saving of the government for its own military consumption and the inestimable advantage to many forms of industry and consequently to labor. If, following an entirely different line, we attempt, either by arbitrary interference with earnings, or by advice as to utilization of land, to reduce food prices by way of the farmer, we find that the same machinery of control would apply equally well to wool and other products not of the nature of food, either in the sense of automatic action, or of an extension of the mechanism of control beyond food stuffs. The saving in woolen clothing alone would equal the entire amount spent in a year for most groups of food stuffs, aside from meats and dairy products, the total for fruits or vegetables.

We have taken it for granted that the government means business in regard to food control and will depart from the precedent of non-interference with business methods. With this assumption, it is worth while to point out that reduction of prices should apply not only to the direct needs of the very poor but of those who will be called upon to bear a good share of the direct expense of the war and upon whom the welfare of the very poor largely depends. Prices should be controlled wherever a simple mechanism can be applied easily and directly to any great necessity or commodity or service of wide use, whether theoretically a necessity or not and it is better to curtail the profits of those of great wealth rather than of those of moderate wealth. Control should certainly include some of the great food staples but simply because these conform to the general principles which make control advisable and we should cut loose from the preconceived notion that what we eat is, under complicated civilized conditions, any more adapted to paternalism than anything else which the public uses in large measure.

